# Small molecules with antiviral activity against the Ebola virus

**DOI:** 10.12688/f1000research.6120.1

**Published:** 2015-02-09

**Authors:** Nadia Litterman, Christopher Lipinski, Sean Ekins

**Affiliations:** 1 Collaborative Drug Discovery, 1633 Bayshore Highway, Suite 342, Burlingame, CA, 94010, USA; 2 Christopher A. Lipinski, Ph.D., LLC., 10 Connshire Drive, Waterford, CT, 06385-4122, USA; 3 Collaborations in Chemistry, 5616 Hilltop Needmore Road, Fuquay Varina, NC, 27526, USA

**Keywords:** Ebola Virus, FDA approved drugs, Medicinal chemistry

## Abstract

The recent outbreak of the Ebola virus in West Africa has highlighted the clear shortage of broad-spectrum antiviral drugs for emerging viruses. There are numerous FDA approved drugs and other small molecules described in the literature that could be further evaluated for their potential as antiviral compounds. These molecules are in addition to the few new antivirals that have been tested in Ebola patients but were not originally developed against the Ebola virus, and may play an important role as we await an effective vaccine. The balance between using FDA approved drugs versus novel antivirals with minimal safety and no efficacy data in humans should be considered. We have evaluated 55 molecules from the perspective of an experienced medicinal chemist as well as using simple molecular properties and have highlighted 16 compounds that have desirable qualities as well as those that may be less desirable. In addition we propose that a collaborative database for sharing such published and novel information on small molecules is needed for the research community studying the Ebola virus.

## Introduction

Viruses remain a constant threat to global health, with new infections from Human Immunodeficiency Virus (HIV), Hepatitis B Virus (HBV) and Hepatitis C Virus (HCV) killing more than 3 million people annually ^1, 2^. The flavivirus that causes dengue fever infects up to 100 million people each year, leading to death in 2.5% of cases ^3, 4^. Other viral outbreaks including severe acute respiratory syndrome coronavirus (SARS-CoV) and the Middle East respiratory coronavirus (MERS-CoV), affect far fewer people but have high mortality rates and the potential to spread to epidemic size ^1, 5, 6^. Thus, even with the development of vaccines and other treatments, viruses lead to a large burden on human health.

More than 30 small molecule drugs have been developed that have activity against individual viruses, including HIV, influenza, HBV, and more recently HCV ^7, 8^. However, a large number of virus types remain without any effective therapeutics, and there are few broad-spectrum anti-virals available. Thus, when viruses emerge that cause life-threatening infections, such as the recent Ebola virus epidemic in West Africa, there are no treatment options. Since it is likely that these and other types of infectious agents will emerge in the future, an important goal is to identify inhibitors to be available to contain such outbreaks.

A large array of drug discovery efforts have proven that despite their small size, viral genomes represent suitable targets for drugs. Direct-acting antivirals, which target viral proteins rather than the host’s, have been the subject of extensive investigation ^3^. Targets in this class fall into multiple categories: virus adsorption inhibitors, inhibitors of viral DNA or RNA synthesis, viral protease inhibitors required for virus maturation, and viral neuraminidase inhibitors required for virus elution ^7^. In addition, cellular targets exist that are required for viral replication, including inosine monophosphate (IMP) dehydrogenase, which is required to supply the pool of guanosine triphosphate (GTP) that serves as a substrate for RNA and DNA, and S-adenosylhomocysteine (SAH) hydrolase, which is required for the methylation and hence maturation of viral DNA. While finding specific inhibitors to target each viral threat individually may be ideal, the cost-savings and feasibility of finding drugs that act as broad-spectrum antivirals, or those that target specific viral genus or family, is an important goal given the expense associated with developing any one drug for one disease ^3^. There are important individual aspects of each virus, and viral family to consider, but nonetheless, many mechanisms of the viral life cycle are mirrored across families, and thus represent opportunities to learn from the many experimental studies that have already been performed.

Like many around the world, we have been watching the devastating effects of the Ebola virus in West Africa. We have been impressed by the important contributions of the health organizations and the personal sacrifices the medical workers are making to serve patients and hamper the spread of disease. And yet we began to wonder, why has there been relatively little focus on small molecules apart from a recent review of Ebola virus therapeutic strategies in general ^9^? Small molecules have several advantages over other therapeutic approaches including the ability to be produced at a large scale and stability necessary for broad distribution. We felt it was time to therefore focus more on small molecules while we await a vaccine. We have found that indeed there is much prior knowledge regarding small molecules that have been shown to be active against the Ebola virus *in vitro* or in animal models ^10– 13^, including a number of FDA-approved drugs ^14– 16^. A thorough literature search of PubMed, and CAS SciFinder ^TM^ (CAS, Columbus OH) using terms including “Ebola” identified 55 molecules suggested to have activity against Ebola virus *in vitro* and/or *in vivo* ( Supplemental Table 1).

### FDA approved small molecules with activity against the Ebola virus

Recently, a pharmacophore ^17^ was generated from four FDA approved compounds for other diseases (non-antivirals) resulting from two high throughput screens against the Ebola virus ^14, 15^ and closely matched the receptor-ligand pharmacophores for the Ebola Viral protein 35 (VP35) ^10^. Follow-up docking studies suggested that these compounds may have favorable inhibitory interactions with this receptor. VP35 is a cofactor in the RNA polymerase transcription complex, and helps the virus evade the immune response by blocking activation of the interferon regulatory factor 3, which is required for the induction of interferons alpha and beta. Thus, blocking VP35 should allow for an enhancement of the host immune response to the Ebola virus. It is proposed that similar compounds may be acting via a closely related mechanism, though there has been no experimental evidence to directly prove this yet.

Another recent study ^16^ has highlighted the ability of three clinically approved ion channel blockers to inhibit the Ebola virus cellular entry. The drugs amiodarone, dronedarone, and verapamil, were given at concentrations that are possible in human serum, and were effective against a number of filoviruses. The authors hypothesized that these drugs may act by disrupting late endosomal processing or by disrupting calcium signaling that is required for viral entry.

Of course, none of these aforementioned FDA approved drugs were designed to target the Ebola virus. Amodiaquine and chloroquine are antimalarials, clomiphene and toremifene are selective estrogen receptor modulators. Amiodarone, dronedarone, and verapamil are anti-arrhythmics. Interestingly, all of these compounds have a common tertiary amine feature, which may suggest they could act through similar mechanism ^18, 19^. However, they are all orally bioavailable and generally safe for humans. Thus these repurposed drugs may represent a fast track to potential evaluation and approval as a feasible option for preventing the spread and mortality associated with the Ebola virus in a large population.

### Small molecules tested in humans with the Ebola virus

Several small molecules have actually been tested in very small numbers of humans for activity against the Ebola virus. For example there has been some press on favipiravir, which is undergoing phase 3 clinical trials in the US for influenza and is approved in Japan, as it has shown promising efficacy against the Ebola virus in mice ^20^. Faviparavir is thought to act by inhibiting the viral RNA-dependent RNA polymerase selectively and has demonstrated activity against a number of other viruses. At least one Ebola patient, who has since recovered, was given favipiravir ^21^, and Japan offered to supply it to the World Health Organization.

A second experimental drug, brincidofovir ^22^, in phase 3 clinical trials for treatment of cytomegalovirus and other DNA viruses has shown efficacy against the Ebola virus *in vitro* and animal studies are ongoing ^23^. Brincidofovir is thought to mimic cytidine, a building block of DNA, and thereby inhibit viral DNA polymerases, and its mechanism of action against the Ebola virus, an RNA virus, is yet unknown. Brincidofovir, which has demonstrated safety in humans, has been given to at least two Ebola virus patients, one in Dallas and one in Nebraska. While unfortunately the Dallas patient died, the Nebraska patient survived ^24^. It is of course too early to know the effect of this molecule on the progression of the disease. This compound is a pro-drug that is converted into the active antiviral, cidofovir diphosphate. Brincidofovir has higher oral bioavailability, intracellular concentrations of drug and increased antiviral potency ^22^. This compound only appeared in the literature in 2014 and there is very little published information.

Beyond these early stage drugs, there are a number of other compounds that have been identified as active against the Ebola virus as summarized by Erik De Clercq ^9^. While many are not ready for in human use, they may present an attractive starting point to be refined in a future drug discovery effort. For example, a novel nucleoside analog, BCX4430 demonstrated efficacy in mice and non-human primates against the Ebola virus ^25^. This compound targets viral RNA polymerase activity by inducing early termination of transcription and thus blocking replication. BCX4430 is not only active against Ebola virus, but also targets other members of the Filovirus family as well as 8 other RNA virus families. Because of the potent and efficacious effects, BCX4430 is being fast-tracked for clinical trials in humans.

### Medicinal chemistry analysis of small molecules active against the Ebola virus

We have recently described an expert’s medicinal chemistry ^26^ analysis of the over 320 NIH probe compounds using public and commercial sources of chemical structures and the issues related to doing this type of analysis ^27^. The likely chemistry quality of these probes was scored based on a number of criteria including literature related to the probe and potential chemical reactivity. Through a series of machine learning models, we also computationally predicted the scores which were being identified through a painstaking manual process ^26^. External validation and comparison with other measures of drug-likeness and filtering rules suggested a comparable level of accuracy ^26^.

We have now carefully analyzed in a similar manner the 55 small molecules with activity against the Ebola virus identified from our literature search. The chemist’s (C.A.L.) decisions on compound quality are summarized in Supplemental Table 1. In contrast to the previous work in scoring compounds as chemical probes, the current aim is very specific - to treat a very serious viral disease for which there is a phenotypic readout. All the known drugs in clinical use were rejected as the chemist looked for compounds that looked more interesting in his opinion. Only 16 out of 55 were selected as desirable in this analysis with no potential problems based on medicinal chemistry experience. In addition to this manual approach, we applied computational filters such as Pan Assay INterference compoundS (PAINS) to identify potentially problematic compounds from structures ^28, 29^ ( Supplemental Table 1). PAINS analysis was enabled using the MMDS mobile app ^30^. Based on this approach we have identified several molecules that appear problematic and agreed with the medicinal chemistry analysis. For example 4 molecules appear to fail the PAINS filters, including the rhodanine compound LJ-001 which was found to be active against numerous enveloped viruses and was found through a screen of inhibitors of Nipah virus entry (IC _50_ 1μM) ^31^. *In vivo* a steady state plasma concentration could not be maintained at a therapeutic level. Rhodanines are known to be problematic PAINS compounds ^28, 29^. Interestingly amodiaquine was not scored favorably by PAINS or the medicinal chemist, yet this is a successful antimalarial drug.

In addition we analyzed the simple chemical properties (calculated in the Collaborative Drug Discovery (CDD) Vault (Collaborative Drug Discovery, Inc) using the Chemaxon toolkit (Chemaxon, Budapest, Hungary)) of the molecules and compared these to their medicinal chemistry classification ( Table 1). The mean calculated molecular property values for compounds were compared using the *t-*test and ANOVA with JMP v. 8.0.1 (SAS Institute, Cary, NC). Significant differences were noted in LogP and Lipinski Rule of 5 violations between desirable and undesirable compounds although one molecule SARA-133 skewed the data with a molecular weight over 3000 ( Table 1). Removal of this compound leads to significant differences for molecular weight, number of hydrogen bond donors, heavy atom count and polar surface area, in addition to logP and Lipinski Rule of 5 violations for desirable compounds versus undesirable compounds.

**Table 1.** Mean ± SD molecular properties calculated in CDD Vault using ChemAxon software for the 55 molecules with activity against the Ebola virus. * statistically significant p < 0.05 using the t-test and ANOVA. ** statistically significant p < 0.0001 using the t-test and ANOVA. Note data are skewed by SARA-133. When this molecule is removed the mean values and significance data are shown in parentheses.

	Molecular weight	LogP	H-bond donors	H-bond acceptors	Lipinski Rule of 5 violations	pKa	Heavy atom count	Polar Surface Area	Rotatable bond number
Undesirable (n = 39)	508.49 ± 447.43 (438.66 ± 101.47)	3.75 ± 4.15 (4.35 ± 1.79)	2.38 ± 5.04 (1.60 ± 1.35)	6.33 ± 10.49 (4.68 ± 2.04)	0.69 ± 0.73 (0.63 ± 0.63)	7.45 ± 4.22 (7.34 ± 0.64)	35.79 ± 30.78 (31.00 ± 7.24)	104.03 ± 197.71 (72.78 ±32.23)	9.67 ± 14.07 (7.47 ± 3.29)
Desirable (N = 16)	371.38 ± 107.47 (*)	1.22 ± 2.55* (**)	3.19 ± 1.94 (*)	5.25 ± 1.77	0.31 ± 0.48* (*)	8.27 ± 3.33	26.06 ± 7.47 (*)	103.36 ± 32.81 (*)	6.37± 6.04

### Collaboration for the Ebola virus drug discovery

**Figure 1.**
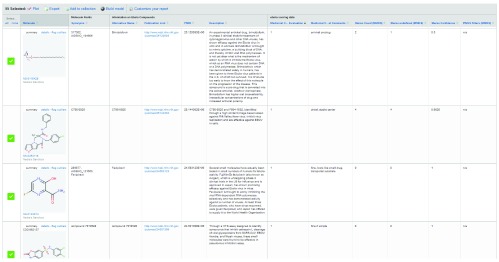
An example of small molecules active against Ebola virus data in the CDD Vault. This illustrates how a comprehensive database could be created and used for collaborations. 55 molecules with predicted properties and scores from an experienced medicinal chemist and filters and shown alongside the publication link and target information.

The research described for small molecule inhibitors against the Ebola virus has occurred in a disconnected manner and there have been few efforts to summarize the total medicinal chemistry efforts to date. We think this research can learn from other areas in which there are currently efforts to improve collaboration and screening. In the area of Tuberculosis research, funding from the Bill and Melinda Gates Foundation and The European Commission have enabled the TB Drug Accelerator and the More Medicines For Tuberculosis, as large-scale collaborations between academia, research institutes and industry. These collaborations have promoted the selective sharing of related data in a secure environment between collaborators using the CDD Vault ^32^ ( Figure 1). The centralized availability of publicly available data on compounds screened for activity against *Mycobacterium tuberculosis* enables researchers to leverage the existing literature alongside their private data. This knowledge can be used for building of validated computational models that can help in selecting additional compounds or lead optimization ^33, 34^, saving time and effort. By organizing the data on small molecules tested against the Ebola virus similarly in a central database and using machine learning models based on public data may help identify additional compounds for testing. Such an effort may also prevent duplication of efforts as we have seen with the screening of multiple libraries of FDA approved compounds against the Ebola Virus ^14– 16^. In order to catalyze this we have made the 55 compounds ( Supplemental Table 1) freely available as a dataset in CDD Public ( https://app.collaborativedrug.com/register). The compound representation in CDD Vault can be accessed via the text compound descriptors (as in the Supplemental Table 1) or in a batch connection table format as an MDL format structure data file (*.sdf) that is universally read by all chemistry aware software. Being able to easily retrieve the chemical structures in machine retrievable form has considerable value. Identifying compounds by name or company code number does not per se allow direct access to chemical structure and often fails entirely to link compound identifier with the chemistry structure of the compound. InChIKey has the great advantage of being searchable on the web (e.g. via Google) but requires a lookup table (e.g. EMBL’s Unichem) to get back to the chemical structure. SMILES and InChi representations allow direct access to chemical structure and are compatible with structure searching in most public chemistry databases as well as the proprietary chemistry ACS CAS SciFinder database. The IUPAC name is universally used in naming chemical compounds in patents and software exists for going from IUPAC name to chemical structure.

## Conclusions

The compounds which are FDA approved drugs for other diseases ^14– 16^ but with activity against Ebola virus *in vitro* or *in vivo* may represent useful starting points with the advantage that much is known regarding their ADME and Tox properties. However they may not be ideal in the opinion of a medicinal chemist. By bringing together all 55 compounds described in the literature with activity against the Ebola virus, this body of evidence may be more convincing than each study in isolation. In addition it allows us to consider structural features and molecular properties, which may provide insights into the target or mechanism of action. It is unclear whether any of the 55 compounds might also have activity when used as combination therapy as is the current standard of care for HIV, in order to overcome drug resistance, and this needs experimental evaluation. Clearly, we are a considerable distance from having an FDA approved drug for the Ebola virus, but this analysis illustrates that there are already drugs on the shelf that can be potentially repurposed in a shorter time period and at a lower cost and yet they do not appear to have been tested in patients with the Ebola virus. In addition there are at least 16 molecules which in the opinion of an experienced medicinal chemist are possibilities for further optimization. While novel compounds are likely more commercially viable they also will require considerable effort to assess safety. Resources will need to be allocated to this effort and administrators and scientists should perhaps consider some of the medicinal chemistry insights we have provided as well as using a collaborative database to share molecules that are active amongst all scientists. It is hoped these efforts could inspire further drug discovery efforts around small molecules.

To illustrate the level of interest in repurposing efforts for the Ebola virus, the following studies were identified upon submission that describe additional compounds as well as those already described herein ^35– 37^.

## Supplementary material

**Supplemental Table 1.** 55 compounds identified from the literature as *in vitro* or *in vivo* active against the Ebola virus. Molecule structures in various 2D formats are provided along with simple molecular descriptors, information relating to the target if known, indication, publication reference as well as medicinal chemistry insights and PAINS filter failures.

http://dx.doi.org/10.5256/f1000research.6120.s42952
