# Supplementary material for: Small molecules with antiviral activity against the Ebola virus
Source: F1000Res. 2015 Feb 9;4:38. [Version 1] doi: 10.12688/f1000research.6120.1 (PMC4335594; doi:10.12688/f1000research.6120.1)
Supplement: Peer review history

# Peer review: Referee response for version 1

**DOI:** 10.5256/f1000research.6555.r7713

**Published:** 2015-02-19

**Authors:** Paul S Anderson

**Affiliations:** 1 Independent Pharmaceuticals Professional, Lansdale, PA, 19446, USA

The authors have collected 55 small molecules reported to have activity against the ebola virus from the literature and organized them into a dataset that is easily searchable for those who may be interested in pursuing further research on the design of inhibitors of this virus.

I have read this submission. I believe that I have an appropriate level of expertise to confirm that it is of an acceptable scientific standard.

# Peer review: Referee response for version 1

**DOI:** 10.5256/f1000research.6555.r7611

**Published:** 2015-02-11

**Authors:** John Lowe

**Affiliations:** 1 JL3Pharma LLC, Stonington, CT, USA

The authors have searched a collection of compounds for a common pharmacophore directed against Ebola proteins, and detected 55 hits. These were filtered by an experienced medicinal chemist using well-established techniques and intuition to provide 16 legitimate compounds that may serve the Ebola research community as a publicly available resource. This is an excellent example of the power of shared, collaborative research databases providing valuable resources to the research community.

I have read this submission. I believe that I have an appropriate level of expertise to confirm that it is of an acceptable scientific standard.
